# Investigation of Ion Irradiation Effects in Silicon and Graphite Produced by 23 MeV I Beam

**DOI:** 10.3390/ma14081904

**Published:** 2021-04-11

**Authors:** Kristina Tomić Luketić, Marko Karlušić, Andreja Gajović, Stjepko Fazinić, Jacques H. O’Connell, Borna Pielić, Borna Radatović, Marko Kralj

**Affiliations:** 1 Ruđer Bošković Institute, Bijenička Cesta 54, 10000 Zagreb, Croatia; Kristina.Tomic@irb.hr (K.T.L.); andreja.gajovic@irb.hr (A.G.); stjepko.fazinic@irb.hr (S.F.); 2 Nelson Mandela University, University Way, Summerstrand, Port Elizabeth 6001, South Africa; 3 Institute of Physics, Bijenička Cesta 46, 10000 Zagreb, Croatia; bpielic@ifs.hr (B.P.); bradatovic@ifs.hr (B.R.); mkralj@ifs.hr (M.K.)

**Keywords:** swift heavy ion, ion track, RBS/c, AFM, TEM, Raman spectroscopy, silicon, graphite

## Abstract

Both silicon and graphite are radiation hard materials with respect to swift heavy ions like fission fragments and cosmic rays. Recrystallisation is considered to be the main mechanism of prompt damage anneal in these two materials, resulting in negligible amounts of damage produced, even when exposed to high ion fluences. In this work we present evidence that these two materials could be susceptible to swift heavy ion irradiation effects even at low energies. In the case of silicon, ion channeling and electron microscopy measurements reveal significant recovery of pre-existing defects when exposed to a swift heavy ion beam. In the case of graphite, by using ion channeling, Raman spectroscopy and atomic force microscopy, we found that the surface of the material is more prone to irradiation damage than the bulk.

## 1. Introduction

Ion implantation is a well-known technological process in the semiconductor industry. This approach of material modification relies on adding large amounts of foreign atoms into the host material, up to several atomic percent, to change its properties. Another approach, investigated in this work, is to utilize the energy of the ion beams to introduce defects into the material and thus influence its properties. In this way, the location, morphology and amount of damage can be fine-tuned by changing ion irradiation parameters (like ion type, energy and fluence) [1,2].

Besides the defect engineering possibilities enabled by this approach, studying the effects of such irradiation is crucial to understanding damage build-up due to irradiation effects in radiation harsh environments, including nuclear and space applications. As a highly important material in the semiconductor industry, silicon has been extensively investigated with regard to resilience to single event upsets [3]. On the other hand, graphite is a very important material in the nuclear industry because it is often used for neutron flux moderation within the nuclear reactor [4]. Therefore, it is essential to understand damage production due to ion irradiation in both materials, and dedicated experiments using accelerator-based techniques are often chosen as the most suitable approach for systematic studies in controlled environments.

Two different energy dissipation channels contribute to ion irradiation effects in materials. At low keV energies, nuclear stopping contributes the most to the damage production due to direct collisions between incoming ions and atoms in the host materials. At higher MeV energies, the nuclear stopping contribution diminishes and eventually becomes negligible. At these high energies, ions can penetrate deep into the material along straight trajectories, with their kinetic energy being lost almost entirely to electron stopping. In this regime, the penetrating ion interacts almost exclusively with electrons within the target material, and produces very dense electronic excitation along its path. Such very localized excitation produced in a nanometer-sized volume and at femtosecond timescale can cause transient melting of the material if the electron-phonon coupling is sufficiently strong, and if the density of deposited energy exceeds a threshold value. This transient molten state typically occurs at the picosecond scale, and it is followed by the rapid quenching that results in a high defect density along the ion trajectory, often referred to as an ion track. Therefore, different regimes of the energy dissipation during ion interaction with the target material lead to different physical processes of damage formation, and distinct damage morphologies. The high energy irradiation, also known as swift heavy ion (SHI) irradiation, produces ion tracks with a characteristic cylindrical structure [5], while the low energy irradiation results in point and cluster defects produced by collision cascades [6].

The aim of this work is to study the response of silicon and graphite to SHI irradiation at the very onset of damage formation due to electronic stopping. This occurs at low MeV energies, when nuclear stopping might not be negligible, and can lead to synergistic effects [7]. Such enhanced damage production can also result in the lowering of the energy threshold for ion track formation, or more generally to any SHI irradiation effect. This interplay of nuclear and electronic stopping in damage formation can be studied in sequential ion irradiation experiments. It is known that low energy ion irradiation (that introduces damage into the material) followed by SHI irradiation, can yield completely different effects. In the case of SrTiO_3_, defects introduced by low energy irradiation sensitize this material to SHI irradiation, effectively lowering the threshold for ion track formation [8], while defects in SiC are effectively annealed after SHI irradiation [9]. 

In the case of silicon, ion beam induced epitaxial recrystallisation (IBIEC) using low energy ions with dominant nuclear stopping has been well-studied [1], but the SHI beam induced epitaxial crystallization (SHIBIEC) has been investigated much less. Although it is practically impossible to produce ion tracks in this material (fullerene beams with extreme values of electronic stopping must be used [10,11]), there is clear evidence that SHI beams can anneal pre-existing damage in silicon [12,13,14]. However, this research has mostly been conducted under various experimental conditions like high values of electronic stopping, elevated temperature, or by using dual ion beams. Therefore, here we investigated the SHIBIEC effect in silicon at room temperature at near threshold electronic stopping power. This should provide useful guidance for future experiments.

An early experimental study has reported the threshold for ion track formation in highly oriented pyrolytic graphite (HOPG) at 7.3 ± 1.5 keV/nm [15], in line with theoretical prediction at 8 keV/nm [16]. Still, the ion tracks in HOPG were unusually small and difficult to model [17], probably due to ion track recrystallisation [15,18], or a large value of electron-phonon coupling [16]. However, due to the possible influence of the velocity effect [19,20], the reported threshold value for ion track formation in HOPG could have been overestimated. Therefore, we report the results of ion track formation in HOPG after exposure to an SHI beam with electronic stopping power below the previously reported threshold [15]. Clearly, the presented results are also of importance for future studies on SHIBIEC in graphite as well because ion track formation can hinder such studies. If damaged material is irradiated by track-forming SHIs, nuclear and electronic stopping contributions to total damage formation could be very difficult to deconvolute. Therefore, it is important to establish a threshold value of electronic stopping power since that would direct SHIBIEC research to SHI beams with electronic stopping below the threshold for ion track formation in HOPG.

## 2. Experimental Methods

In this work, silicon samples used for irradiation were cut from a single crystal silicon wafer with a well-defined and polished <100> surface. The samples were 1 × 1 cm^2^ in size, with thickness of 0.3 mm. No prior sample preparation had been done before the irradiation. The high quality highly oriented pyrolytic graphite (ZYA HOPG) samples were purchased from 2SPI. The samples were 1 × 1 cm^2^ in size and 0.7 mm thick, with a mosaic spread angle of 0.4° ± 0.1°. No surface preparation was done prior to irradiation, except on the sample later used for atomic force microscopy (AFM) analysis that was freshly cleaved with scotch tape.

Irradiation of silicon was done using iodine ion beams with energies of 600 keV and 23 MeV, while the irradiation of HOPG was done using only 23 MeV iodine. These beams were delivered by the 6 MV EN Tandem Van de Graaff accelerator (HVEC, Burlington, MA, USA) located at the Ruđer Bošković Institute (RBI). Irradiations have been done either at normal incidence (5° off normal to avoid the channeling) or at grazing incidence (1° with respect to the surface). All irradiation parameters used in this work are reported in Table 1. 

After irradiation, analysis of silicon and HOPG samples by Rutherford backscattering spectrometry in the channeling mode (RBS/c) was accomplished using a 1 MeV proton beam delivered by the 1 MV Tandetron accelerator (HVEE, Amersfoort, Netherlands) located at the RBI. The analyzing beam spot size was 1 mm, and the beam current was limited to few nA. The samples were aligned on an unirradiated part of the surface by obtaining angular (tilt, azimuth) scan maps. These maps have been acquired by typical 2° × 2° scans and with 20 × 20 pixels resolution, resulting in the channel alignment within 0.1°. For detection of backscattered ions, a silicon surface barrier detector was positioned at 160° with respect to the proton beam direction [21,22].

Cross sectional transmission electron microscopy (TEM) lamellae were prepared by the focused ion beam (FIB) lift-out technique using an FEI Helios NanoLab 650. Prior to exposing the irradiated surface of the silicon specimen to Ga ions, a ~100 nm thick protective carbon layer was deposited using the electron beam. Final polishing of the lamellae was performed at a Ga beam energy of 2 kV. TEM imaging was performed using an image corrected JEOL ARM200F at 200 kV.

The HOPG samples were investigated by Raman spectroscopy within a few hours of irradiation. The spectrometer used was a Horiba Jobin Yvon T64000. For the excitation, a 532 nm solid-state laser with a power of 4 mW at the sample was used with a 50× long working distance objective. 

Atomic force microscope (AFM) images of the HOPG surface were taken with a JPK Nanowizard Ultra Speed AFM under ambient conditions. Non-contact AC (tapping) mode was used for acquisition with a setpoint around 55%. Bruker TESP-V2 silicon tips with a nominal spring constant of 37 N/m, a tip radius of 7 nm and a resonant frequency of 320 kHZ were used. Images were processed with JPK Data Processing software (SPM software v6.0, JPK, Berlin, Germany).

## 3. Results and Discussion

### 3.1. SHIBIEC Effect in Silicon

**Figure 1:**
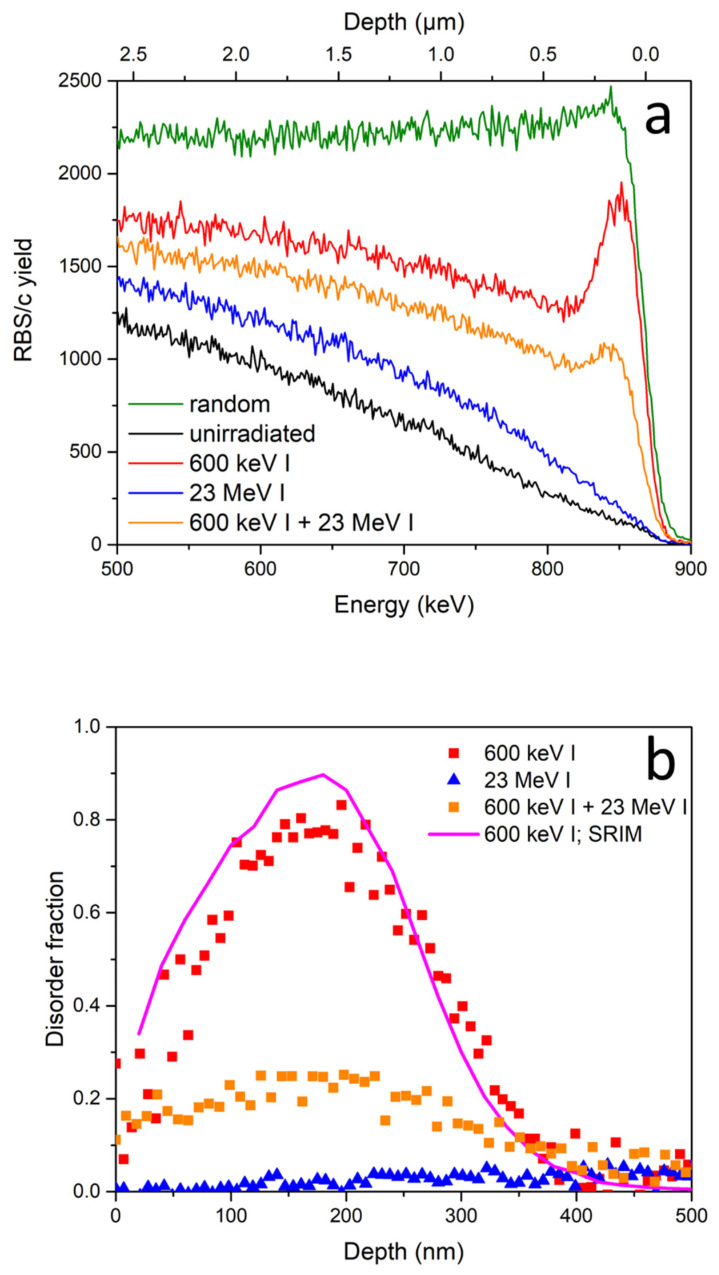

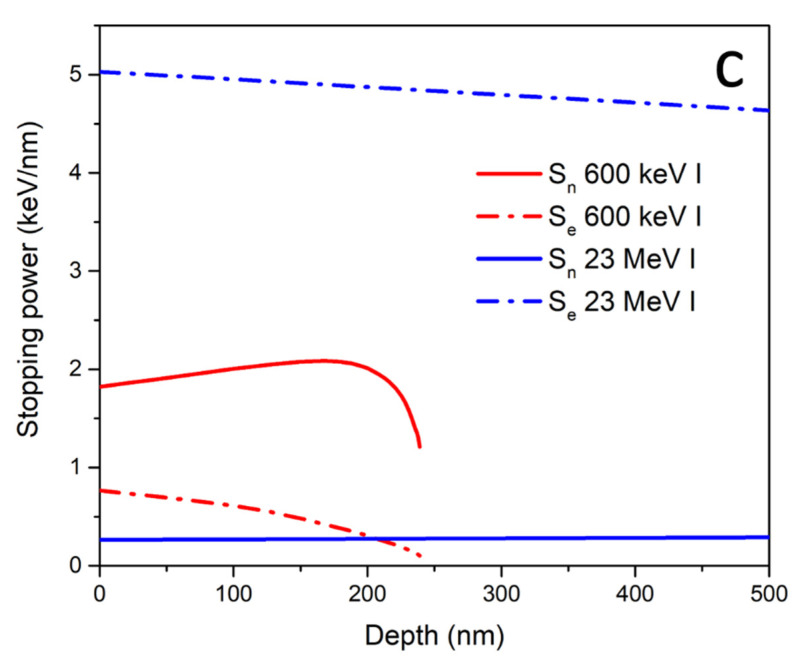
(**a**) RBS/c spectra obtained from the sequentially irradiated silicon sample. Damage in silicon is observed after 600 keV I irradiation up to a fluence of 10^14^ ions/cm^2^ as a pronounced damage peak at proton energies between 830–880 keV. After irradiation of damaged Si with a 23 MeV I beam up to a fluence of 10^14^ ions/cm^2^, recovery of the damage can be observed as a decrease of the same peak. (**b**) Disorder fraction depth profiles obtained by the two-beam approximation method. Additionally, the damage depth profile obtained by SRIM is also given [23]. (**c**) Nuclear and electronic stopping powers of silicon for the iodine projectiles used [23].

First, we present the results of our study on sequential ion irradiation of silicon. As shown in Figure 1a, spectra obtained by RBS/c measurements provide clear evidence of SHIBIEC when using a 23 MeV iodine beam. As expected, after irradiation of pristine silicon with only 23 MeV iodine up to a fluence of 10^14^ ions/cm^2^, only a low concentration of point defects were found that caused slight de-channeling of the probing RBS/c beam. The observed de-channeling cannot be caused by electronic stopping that is well below the threshold for ion track formation in silicon. These defects were most likely produced by the very low, but not negligible nuclear stopping component of the 23 MeV iodine beam. Irradiation with the 600 keV iodine beam to a fluence of 10^14^ ions/cm^2^ produced a shallow but highly damaged layer of material. Analysis of the RBS/c spectra using a two-beam approximation [24] yielded a depth of the damaged layer of ~300 nm, with a disorder fraction of up to 80% as shown in Figure 1b, and in accordance with the TRIM code simulations [23]. Finally, the RBS/c spectrum obtained from the sample area exposed to sequential ion irradiation with 600 keV and the follow-up 23 MeV iodine beam, both to the fluence of 10^14^ ions/cm^2^, shows a much lower amount of damage. This is clearly seen as the height of the observed peak around 850 keV is very much reduced, and quantitative analysis shown in Figure 1b indicates the reduction of the disorder fraction from 80% down to 25%. As shown in Figure 1c, the electronic stopping of the silicon for the 23 MeV I beam is nearly constant over the damaged region, hence reduction of the disorder fraction appears to be proportional to the initial level of disorder.

Disorder fraction was estimated from the observed RBS/c spectra by the two-beam approximation, using an iterative approach [24]. The backscattering yield in channeling originates from the channeled particles backscattered from displaced atoms, and from the dechannelled particles backscattered from any atom in the crystal. The investigated sample is divided into increments of equal thickness in which the disorder fraction is considered constant. Starting with the topmost (surface) layer, the disorder fraction is calculated, based on the differences in the yields of the spectra from the unirradiated and irradiated areas, and this gives the estimate of the probability that an ion becomes dechannelled in the next layer. This probability enables the calculation of disorder fraction in the next layer, which in turn gives the probability of dechanneling in the subsequent layer. The iterative procedure is carried out until depths well below the disordered region have been reached [24].

**Figure 2:**
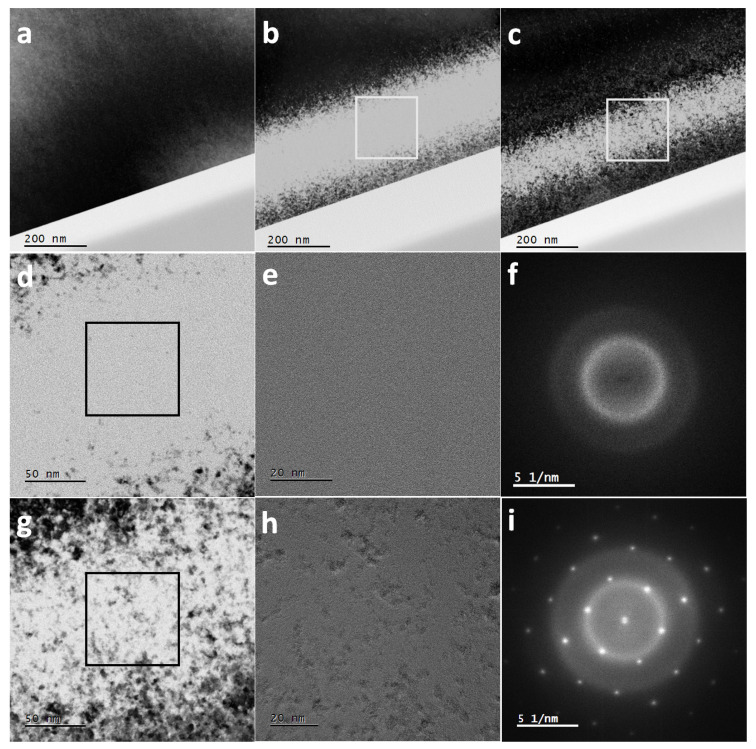
Cross-sectional BF TEM images of silicon irradiated with: (**a**) 23 MeV iodine, (**b**) 600 keV iodine and (**c**) both 600 keV and 23 MeV iodine. Magnified region (**d**) and HRTEM image (**e**) of the damaged zone (denoted by the square in (**b**) and (**d**), respectively), (**f**) corresponding FFT of image (**e**). Magnified region (**g**) and HRTEM image (**h**) of recovered zone (denoted by the square in (**c**) and (**g**) respectively), (**i**) the corresponding FFT of (**h**).

Complementary TEM measurements confirm the recovery of the damage in Si (obtained by 600 keV I irradiation) after the damaged specimen was subsequently irradiated with a 23 MeV I beam (Figure 2). As expected, 23 MeV iodine irradiation shows no ion tracks, and also no discernable damage (a). The image was taken close to a zone axis causing strong diffraction contrast which is rather uniform over the field of view. After low energy (600 keV) iodine irradiation (b), material is severely damaged and the amorphized region no longer produces dark diffraction contrast. After sequential ion irradiation (c), restoration of crystallinity is visible, although not complete. The magnified views of the central part of the 600 keV damage zone (square region in Figure 2b) is shown in Figure 2d, while an HRTEM image of the square region in (d) is displayed in Figure 2e. These images ((d) and (e)), together with the corresponding Fast Fourier Transform (FFT) of the HRTEM image (Figure 2f), which shows no reciprocal lattice spots, clearly indicates a lack of structure after irradiation with 600 keV ions. The magnified image of the sequentially irradiated specimen using a 23 MeV I beam (square region in Figure 2c) is presented in Figure 2g while the HRTEM image is shown in Figure 2h. From the corresponding FFT in Figure 2i only one reciprocal lattice is visible after sequential irradiation with 23 MeV I, which means that the material recrystallizes in the same orientation everywhere, suggesting that some sort of pseudo ordering remains, i.e., the material “remembers” the original orientation. The depth of the damage band is found to be up to around 300 nm, in agreement with TRIM simulations. 

Presented results clearly demonstrate that the SHIBIEC effect is already active in silicon at 5 keV/nm electronic stopping and at room temperature. This is much lower than the 10.5 keV/nm value of the previously used 100 MeV Ag beam [12]. Our results are also in good agreement with a recently reported SHIBIEC study [13], where the weaker effect of electronic stopping was compensated for by much higher applied fluences. Moreover, our results are consistent with another recent dual beam irradiation experiment [14], where both low energy 900 keV iodine and high energy 27 MeV Fe or 36 MeV W beams were simultaneously applied to a silicon target. Although the result was influenced by the flux of the applied ion beams, it was clear that simultaneous irradiation yields less damage compared to irradiation by only low energy ions. Future studies of interest would include investigations both of ion fluence and flux on the observed SHIBIEC in silicon from which single ion effects could be deduced [9].

Also, we note that calculations indicate that amorphous silicon could be sensitive to ion track formation for electronic stopping below 10 keV/nm [25], which is in line with experimental observations [26]. Therefore the present work paves the way for future experiments aiming to answer the open question: how does the amount of pre-damage and other irradiation parameters lead to two distinctive phenomena in silicon, namely ion track formation in completely amorphous silicon and SHIBIEC in moderately damaged silicon?

### 3.2. Ion Tracks in Graphite

Next, we present results of ion track formation in HOPG. Previously, very high energy ions have been used to produce ion tracks in graphite, and these were found to be smaller than tracks in other materials [27]. Since graphite is a sensitive material, mostly scanning probe techniques and Raman spectroscopy have been used to investigate SHI effects in HOPG. In the following, we present our RBS/c, Raman spectroscopy and AFM results on the SHI irradiated HOPG.

**Figure 3:**
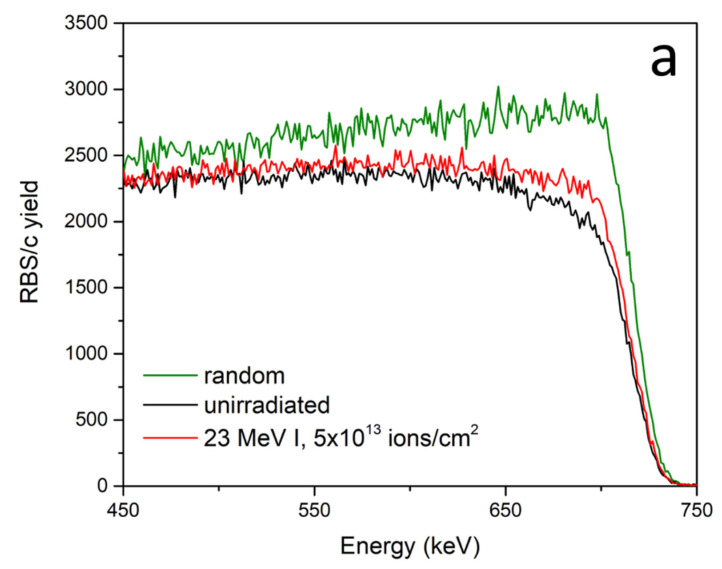

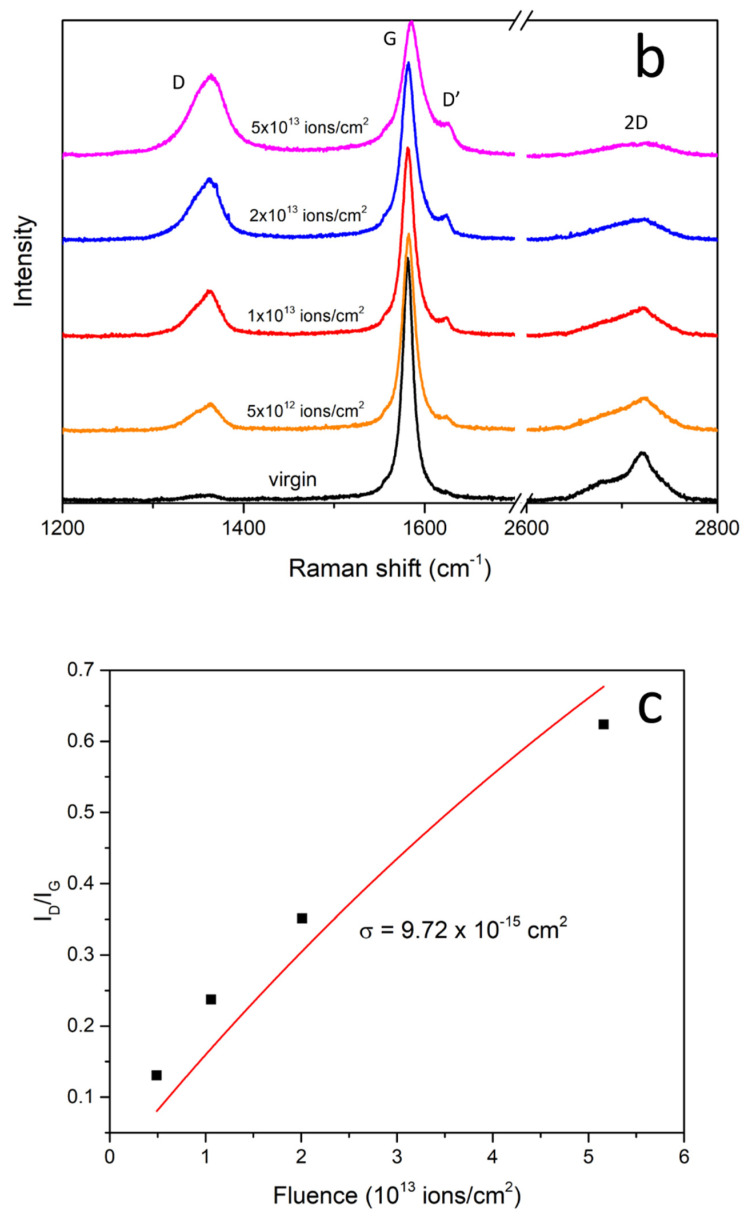
(**a**) RBS/c spectra from HOPG irradiated at normal incidence with 23 MeV I to 5 × 10^13^ ions/cm^2^. (**b**) Raman spectra from HOPG irradiated with 23 MeV I to different fluences up to 5 × 10^13^ ions/cm^2^. (**c**) Damage kinetics in HOPG obtained from Raman spectroscopy data and presented as an increase of I_D_/I_G_ ratio.

Figure 3a shows spectra obtained from RBS/c measurements. The probing beam was 1 MeV protons because spectra obtained with the He beam had a much lower count rate, although the He beam has been used in the past for channeling experiments in HOPG [28,29]. It was possible to achieve channeling in HOPG because the mosaic spread was low, around 0.4°, demonstrating good sample quality. As shown in Figure 3a, irradiation of HOPG with 23 MeV I up to 5 × 10^13^ ions/cm^2^ introduced only a small amount of damage into the material. Considering the yield increase with respect to the virgin and random spectra, the amount of damaged material is estimated to be around 10%. For the given fluence, and considering possible ion track overlap, the radius of the ion track is estimated to be *r* = 0.25 nm. Clearly, these ion tracks are very small and most likely discontinuous in nature [27,30]. In our opinion, a 23 MeV I beam is below the threshold for ion track formation and what we observed is only subthreshold damage consisting of clusters of defects produced along the ion trajectory [31] with a damage cross section of σ~0.2 nm^2^.

Results of the Raman spectroscopy measurements on the 23 MeV I irradiated HOPG are shown in Figure 3b. As Raman spectroscopy is a surface sensitive technique probing the first ~50 nm in graphite [32], a 100 nm thin carbon foil was placed before the HOPG in order to equilibrate the charge state of the ion beam to avoid possible charge state effects [33]. Raman spectra show the evolution of four distinct bands that arise upon the irradiation of graphite with increasing fluence of the 23 MeV I beam. As expected, the virgin sample shows only three prominent bands: the G-band at 1580 cm^−1^ and the 2D-band doublet comprised of two peaks, at 2690 cm^−1^ and 2730 cm^−1^. The G-band is indicative of the relative motion between carbon atoms, while the 2D-band doublet are overtones of the D-band, and are active even without the presence of defects. A negligible bump at a wavenumber corresponding to the D-band is also present in the virgin sample. Starting with the lowest fluence of 5 × 10^12^ ions/cm^2^, in addition to the G- and 2D-bands, two other bands emerged: D-band at 1360 cm^−1^ and D’-band at 1620 cm^−1^. These bands require the presence of defects to become activated [34]. Moving further to the samples irradiated by higher fluences, from 1 × 10^13^ ions/cm^2^ to 5 × 10^13^ ions/cm^2^, a noticeable increase in the intensity of D- and D’-band occurred, while the G-band and 2D-band show discernible decrease of the peak intensities accompanied by peak broadening. A D’-peak, that becomes more prominent with increase of the fluence, can be thoroughly studied in order to determine the type of defects present in the material. Such analysis gives the value of I_D_/I_D’_ ≈ 3.8, which is characteristic for grain boundary type defects, close to the minimum value of I_D_/I_D’_ ≈ 3.5 measured in graphite [35]. The 2D-band retained its doublet structure even for the highest fluence of 5 × 10^13^ ions/cm^2^, despite its significant broadening. 

This modification of the 2D-band is an indication of structural disarrangement of the constitutive six-fold carbon rings, hence this kind of peak evolution in the Raman spectra suggests the first stage of carbon amorphization [34]. In addition to the retained doublet structure of the 2D-band, this suggests, alongside the above determined existence of boundary-type defects, a certain amount of graphite transformed to its nanocrystalline correlative. However, even for the highest ion fluence of 5 × 10^13^ ions/cm^2^, the disorder-induced D-band shows smaller intensity than the G-band, and the I_D_/I_G_ ratio remains less than one. While it is known that for high enough fluences the intensity of the D-band can exceed G-band intensity by a factor of almost two, it was observed that the other SHI beams with energies of several hundreds of MeV are even less effective in ion track production in HOPG [20]. 

To evaluate damage cross section from Raman spectroscopy measurements, ion track overlap at high fluences has to be considered [5,7]. Only for low ion fluences, damage increases almost linearly with the fluence, and this is observed in Figure 3c. For high enough ion fluences, ion impact into already damaged material will not produce further increase in the intensity of D-band. This type of single ion impact damage kinetics is described by the Poisson law [7] and fit to the experimental data is presented in Figure 3c. By considering a saturation value of I_D_/I_G_ at 1.7 [20], we estimated the damage cross section in HOPG from Raman spectroscopy measurements to be σ = 0.97 ± 0.09 nm^2^. This value was larger than the damage cross section obtained from RBS/c measurements, but is also in agreement with previous works where Raman spectroscopy yielded larger ion track sizes than other techniques like TEM [36]. While the TEM and RBS/c measurements of ion track sizes agree very well [37], Raman spectroscopy is sensitive not only to the ion track itself, but also to defects in the ion track halo, as well as strain fields surrounding the ion track [36]. The phase transformations of HOPG into graphene and diamond were observed previously due to irradiations with cluster ion beams and highly charged ion beams, with bands observed at 1300 cm^−1^ and 1443 cm^−1^ designating the transition into nanodiamond [38] and a band observed at 2643 cm^−1^ that indicates the transition into graphene [39]. None of these peaks have been observed in our spectra, indicating that the electronic stopping power which we used was too low to induce any of those phase transformations.

**Figure 4:**
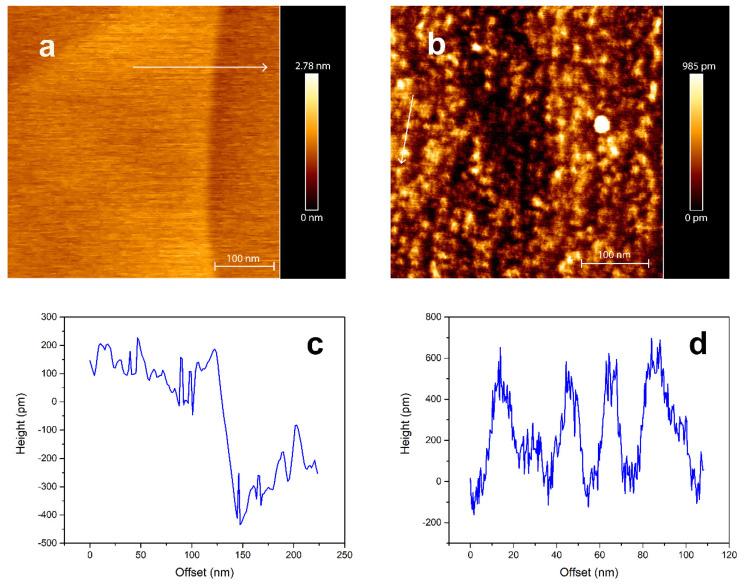
AFM images of (**a**) unirradiated HOPG surface, (**b**) surface irradiated with 23 MeV I at grazing incidence with (**c**) height profile across surface step and (**d**) height profile of the ion track indicated in (**b**).

The irradiation effects on the HOPG surface morphology were also studied. As shown in Figure 4, irradiation of HOPG at a grazing angle of 1° with respect to the surface does produce ion tracks that can be observed with AFM under ambient conditions. Compared to the unirradiated area that exhibits a very flat surface shown in Figure 4a, many ion tracks are visible on the sample area exposed to the 23 MeV I beam (Figure 4b). Figure 4c shows height profile of unirradiated HOPG surface indicated with a white arrow on Figure 4a,d shows height profile of irradiated HOPG surface indicated with a white arrow on Figure 4b. It is visible that unirradiated HOPG has smooth surface with corrugation in range of 100 pm within one terrace and that after irradiation HOPG has visible ion tracks that corrugate surface more than 500 pm. These tracks have a distinct discontinuous morphology [40], that is also observed in other materials [2,41,42,43,44,45] due to the locally varying electronic stopping power experienced by the SHI when passing through atomic layers at grazing incidence. 

It might seem surprising that ion tracks have been observed on the HOPG surface, while the tracks in the bulk have not been confirmed by RBS/c. However, the presence of the surface is known to facilitate ion track formation possibly due to several reasons [44]. For example, oscillating stopping power can exceed the average value of stopping that is related to ion track formation in the bulk material, especially for 5° off-normal irradiation usually done to avoid SHI channeling [46]. Another possible reason is a more efficient recrystallization process in the bulk material, when in some materials melt undergoes partial or complete epitaxial recrystallization [47]. Therefore, the observation of tracks on the HOPG surface after grazing incidence SHI irradiation is not completely unexpected, and results presented here provide evidence that the threshold for ion track formation on the HOPG surface is below 6.7 keV/nm. This is lower than the threshold reported previously at 7.5 keV/nm [15], which might be affected by the velocity effect. For silicon we do not expect to observe surface ion tracks at 5 keV/nm since none were observed previously at a higher stopping power of 12 keV/nm [48].

## 4. Conclusions

We present evidence of SHI irradiation effects in silicon and graphite found after 23 MeV I irradiation. In the case of silicon, RBS/c and TEM measurements provide evidence for the SHIBIEC effect already at an electronic stopping power of 5 keV/nm. This demonstrates the potential of SHI beams for effective defect engineering in silicon, the material that is otherwise known to be extremely resistant to ion track formation. Results obtained by RBS/c, Raman spectroscopy and AFM give proof of ion track formation in HOPG, but only at the surface. The low value of electronic stopping of merely 6.7 keV/nm indicates that the material is more susceptible to damage at the surface than in the bulk, which should be considered when planning to use graphite in radiation harsh environments.

## Figures and Tables

**Table 1:** Irradiation parameters used in this work: ion type and energy, nuclear energy loss S_n_/dx, electronic energy loss S_e_/dx and ion range R, according to the SRIM code [23].

Material	Ion Beam	S_n_/dx (keV/nm)	S_e_/dx (keV/nm)	R (μm)
silicon	600 keV iodine	1.82	0.77	0.23
	23 MeV iodine	0.26	5.03	6.81
	1 MeV protons	0	0.04	16.34
HOPG	23 MeV iodine	0.27	6.72	5.56
	1 MeV protons	0	0.05	12.37

## Data Availability

Data is available on request.
